# Short Tandem Repeats as a High-Resolution Marker for Capturing Recent Orangutan Population Evolution

**DOI:** 10.3389/fbinf.2021.695784

**Published:** 2021-08-16

**Authors:** Alina-Alexandra Voicu, Michael Krützen, Tugce Bilgin Sonay

**Affiliations:** ^1^ Department of Computer Science, ETH, Zurich, Switzerland; ^2^ Department of Anthropology, University of Zurich, Zurich, Switzerland; ^3^ Department of Ecology, Evolution and Environmental Biology, Columbia University, New York, NY, United States

**Keywords:** short tandem repeats, genetic variation, orangutan, population diversity, local adaptation, recent evolution

## Abstract

The genus *Pongo* is ideal to study population genetics adaptation, given its remarkable phenotypic divergence and the highly contrasting environmental conditions it’s been exposed to. Studying its genetic variation bears the promise to reveal a motion picture of these great apes’ evolutionary and adaptive history, and also helps us expand our knowledge of the patterns of adaptation and evolution. In this work, we advance the understanding of the genetic variation among wild orangutans through a genome-wide study of short tandem repeats (STRs). Their elevated mutation rate makes STRs ideal markers for the study of recent evolution within a given population. Current technological and algorithmic advances have rendered their sequencing and discovery more accurate, therefore their potential can be finally leveraged in population genetics studies. To study patterns of population variation within the wild orangutan population, we genotyped the short tandem repeats in a population of 21 individuals spanning four Sumatran and Bornean (sub-) species and eight Southeast Asian regions. We studied the impact of sequencing depth on our ability to genotype STRs and found that the STR copy number changes function as a powerful marker, correctly capturing the demographic history of these populations, even the divergences as recent as 10 Kya. Moreover, gene ontology enrichments for genes close to STR variants are aligned with local adaptations in the two islands. Coupled with more advanced STR-compatible population models, and selection tests, genomic studies based on STRs will be able to reduce the gap caused by the missing heritability for species with recent adaptations.

## Introduction

Wild orangutans inhabiting the islands of Sumatra and Borneo are the only Southeast Asian great apes (Wich et al., 2008). They belong to the *Pongo* genus, diverged into three distinct species, the Sumatran *Pongo abelii* and *P. tapanuliensis* and the Bornean *Pongo pygmaeus* (Wich et al., 2008; Nater et al., 2017). The demographic history of orangutans has been shaped by dramatic changes in climate and sea levels (Flenley, 1998). A recent study (Mattle-Greminger et al., 2018) investigating the demographic history of orangutans found strong support for a scenario in which orangutans from mainland Asia migrated to the south of present-day Lake Toba in Sumatra. Descendants of this initial population subsequently colonized other regions in Sundaland. The population found in the north of Lake Toba deeply diverged from the one found in the south of Lake Toba approximately 3.4 Ma ago, forming the recently identified species *P. tapanuliensis* (Nater et al., 2017). Orangutans from southern Sumatra migrated to Borneo 1 Ma to 700 Kya (Mattle-Greminger et al., 2018), their transition being facilitated by low sea levels and elevated ice volume during the glaciation periods in the Pleistocene epoch. According to the demographic model, the Bornean population experienced a bottleneck period 110–30 Kya characterized by increased gene flow within the population, which indicates that the orangutans retreated to a common region. The bottleneck was followed by an expansion in the Bornean population 30–10 Kya, followed by the divergence of the three Bornean subspecies: *Pongo pygmaeus*, *Pongo pygmaeus morio* and *Pongo pygmaeus wurmbii*.

Once widely spread throughout mainland Southeast Asia and Sundaland, the orangutans are nowadays encountered exclusively in isolated regions in northern Sumatra (the *Pongo abelii* and *P. tapanuliensis* species) and in Borneo (the *Pongo pygmaeus* species) (Wich et al., 2008). Since their divergence, these species have been subjected to contrasting environmental factors (Taylor and van Schaik, 2007; Wich et al., 2009). On one hand, the Sumatran population benefited greatly from a stable environment, characterized by relatively constant food supply and moderate climate (Wich et al., 2006; Wich et al., 2011). On the other hand, the Bornean population was faced with severe, fluctuating environmental factors and food scarcity as a result of the El Nino phenomenon (Arora et al., 2010; Wich et al., 2011). These highly disparate conditions left an imprint on each of the populations’ genetic heritage, prompting the orangutan populations to exhibit highly distinct phenotypes, namely different morphology, behavioral patterns, and social structure. The Sumatran population shows evidence of the selection of genes associated with high-level cognitive functions, whereas the Bornean population, urged to become better equipped for survival, shows signs of selection of metabolism- and energy-related genes (Mattle-Greminger et al., 2018).

Understanding genomic variation is pivotal to our grasp of population history. Studies of genomic variation on wild great ape species rely primarily on single nucleotide polymorphisms (SNPs) (Locke et al., 2011; Prado-Martinez et al., 2013; Mattle-Greminger et al., 2018). SNP markers have been regarded as the most viable tools for characterization of natural populations due to their homogeneous mutation load as well as their suitability to the advances in next generation sequencing along with the numerous tools and statistical approaches that shaped how we study population genetics in the last decade (Yang et al., 2010; Altshuler et al., 2012; Khan et al., 2012).

However, recent studies analyzing patterns of human genetic variation, suggested that population studies based on SNPs might fail to capture a considerable amount of variation, which can limit the power of genome-wide association studies to detect biologically relevant signals. This has been deemed the *‘missing heritability link’* (Manolio et al., 2009; Gibson, 2010). One class of rare variants that may close this gap is indels (insertions and deletions). These variants are being increasingly recognized due to their abundance and multi-allelic nature (Montgomery et al., 2013; Elizabeth & Biolabs, 2015). *Alu* insertion polymorphisms were, in fact, among the first evolutionary markers in early population studies before the genomic era, contributing to uncover the African origins of modern humans (Stoneking et al., 1997; Nasidze et al., 2001). With the revolutionary changes in high throughput technologies, researchers expanded their datasets from a few loci to genome-wide markers, increasing the statistical power in population analyses. Studies based on thousands of indel loci yielded novel variants associated with complex traits, and population structure that are not linked to SNPs (Montgomery et al., 2013; Gudbjartsson et al., 2015).

Unlike SNPs, indels do not exhibit a homogeneous mutation load, which required further classification and studying those classes separately (Montgomery et al., 2013). An abundant class of indels, which holds the promise to explain an important portion of the missing heritability link in recent evolutionary history is microsatellites or the short tandem repeats (STR) (Press et al., 2014).

Previously disregarded as junk DNA, STRs are repetitive DNA sequences whose building blocks are short, recurring DNA motifs of two–six nucleotides (Li et al., 2002). The repetitive structure of a short tandem repeat prompts polymerase slippage events, which trigger copy number changes of STR’s constituent motifs, namely expansions and contractions during cell divisions (Legendre et al., 2007). These copy number mutations occur at a rate of 10^*–*2^ to 10^*–*6^ per cell division, which is 100–10,000 times higher than point mutations (Willems et al., 2014; Mousavi et al., 2019). STRs’ high mutational rate as well as their frequent reverse mutations complicates tracing the evolution of ancient divergences. On the other hand, for the recent evolutionary events, due to their multi-allelic spectra, STRs can be more informative compared to SNPs, which have a bi-allelic character. Their highly dynamic and diverse mutational patterns present a tremendous variation between recently diverged populations, making them ideal candidates for studying recent evolution (Gemayel et al., 2010).

Although population studies based on a few loci frequently used microsatellite markers, which were once regarded as *the* standard genetic marker (Jorde et al., 2000; Coates et al., 2009), until recently they were largely disregarded from large-scale analyses of genetic variation due to the challenges in their sequencing and genotyping (Guilmatre et al., 2013; Mousavi et al., 2019). High quality PCR-free genomic sequencing as well as STR compatible mapping approaches surmounted the issue of low confidence, increasing the concordance with capillary data up to 98.5% (Gymrek et al., 2012; Willems et al., 2017; Mousavi et al., 2020) and 100% with Sanger sequencing (Rocca et al., 2020). With these advancements, it was shown that short tandem repeats correctly capture known population diversity patterns in humans (Willems et al., 2014) and primate species (Bilgin Sonay et al., 2015).

Next to their contribution to our understanding of population structures, hence in genotypic variation, STRs are widely studied for their role in phenotypic variation. They are abundant at gene regulatory regions and play a role in gene expression divergence, especially when they are located in very close proximity to genes, such as promoters (Gymrek et al., 2012, 2015; Bilgin Sonay et al., 2015; Quilez et al., 2016; Fotsing et al., 2019; Sulovari et al., 2019). A well-studied example comes from polymorphic short tandem repeats found in the promoter region of the prolactin one gene that regulate the adaptation of *Tilapia* fish to various salinity levels (Streelman and Kocher, 2002).

Here, we explore for the first time whether STRs manage to capture established genetic diversity patterns in wild orangutan populations. Our study capitalizes on an extensive genomic dataset which encompasses 21 publicly available wild orangutan genomes with known provenance covering the entire range of the *Pongo* genus with individuals from four sampling sites each in Sumatra and in Borneo (Prado-Martinez et al., 2013; Mattle-Greminger et al., 2018). Based on our comprehensive STR panel, we explore the genetic diversity of the Sumatran and Bornean populations and trace the genomic origins of their adaptive history.

## Methods

### Dataset

Our dataset contains the whole-genome sequencing data of 21 orangutans from the Southeast Asian islands of Sumatra and Borneo and encompasses the entire range and subdivisions of the *Pongo* genus (see Supplementary Table S1). This represents a subset of the 37 genomes previously investigated in a recent study aimed at the elucidation of the adaptive evolution of wild orangutans (Mattle-Greminger et al., 2018). We restricted our analysis to genomes with at least 10x coverage to ensure quality in the reads we considered.

The dataset comprises 12 individuals from the Sumatra island belonging to the *Pongo abelii* (*n* = 11) and *P. tapanuliensis* (*n* = 1) species and nine individuals from the Borneo island belonging to the *Pongo pygmaeus* (*n* = 3) and *Pongo pygmaeus morio* (*n* = 6) subspecies. Our panel includes individuals which originate from four Sumatran sampling sites: Langkat (*n* = 4), North Aceh (*n* = 2), West Alas (*n* = 5), and Batang Toru (*n* = 1), as well as from four Bornean sampling sites: Sarawak (*n* = 3), East Kalimantan (*n* = 2), North Kinabatangan (*n* = 2), and South Kinabatangan (*n* = 2).

### Identification of Short Tandem Repeats in the *Pongo abelii* Reference Genome

The reference genome for genus *Pongo* is the Sumatran *Pongo abelii* genome (PonAbe2). In order to identify the STRs in the reference genome, we used the Tandem Repeats Finder (TRF) software version 4.09, whose implementation is based on the Smith Waterman dynamic programming algorithm which aligns two sequences against one another (Benson, 1999). This program takes as input the DNA sequence of each chromosome in the FASTA format. We generate the STR catalog in the reference genome using the set of parameters as recommended, namely MATCH = 2 (score assigned when two aligned nucleotides match), MISMATCH = 7 (penalty for the mismatch of two nucleotides in the alignment), ‘DELTA = 7 (penalty for insertions or deletions), PM = 80 (matching probability), PI = 10 (indel probability), MIN SCORE = 14 (minimum TRF score for an STR to be reported). An indel probability of at most 10% means, for instance, that a STR with 10 copies of a trimeric motif can have in total at most three inserted or deleted nucleotides relative to the neighboring copies. A matching probability of at least 80% means, for example, that an STR with 10 copies of a dinucleotide motif must contain at least 16 out of 20 nucleotides which are identical to the adjacent pattern. We set the minimum threshold for the alignment score of the tandem repeats in our STR catalog to be 14, as recommended in (Bilgin Sonay et al., 2015). To illustrate how the TRF score is computed, we provide the following example: under the assumption of a perfect alignment (no mismatches, no deletions, no insertions), an STR with seven copies of the GC motif would be assigned a TRF score of 14, and it would therefore be reported by TRF.

We filtered the STRs identified by TRF by selecting only those which fulfill a number of quality criteria. First, we selected the tandem repeats whose repeat unit length is comprised between two and six bps, as we are interested in short repeats due to their especially high polymorphism (Willems et al., 2014). Furthermore, we excluded the STRs whose total length is more than 100 bps. We imposed this restriction since genotyping algorithms require that an STR is fully encompassed within any short read, which is 100 bps long in the current short-read technologies. For the STRs whose repeat unit is 2, 3, 4, 5 or 6 bps, we required that their total length is at least 13, 20, 23, 27, or 27, respectively based on Fondon et al.’s definition of STR (Fondon et al., 2012). In case of overlapping repeats, we chose repeats with the greatest scores that are at least 20 bps apart. We excluded STRs which overlap with transposons, telomeres and centromeres since their sequence contain low-fidelity portions (Lamb and Birchler, 2003; Aldrup-MacDonald and Sullivan, 2014; Miga, 2015) and are subject to different mutational forces than STRs (Munoz-Lopez and Garcia-Perez, 2010; Saint-Leandre et al., 2019). The coordinates of the telomeres, centromeres, and transposons were retrieved from the UCSC browser (Kuhn et al., 2013). Our reference STR catalog comprises 436059 STRs.

### Building the Catalog of Wild Orangutan Short Tandem Repeat Variation

In order to genotype STRs in each of the orangutan genomes in our dataset, we used lobSTR, a C++-based tool developed for the profiling of STRs in personal genomes (Gymrek et al., 2012). The alignment and the allelotyping steps were run on one genome at a time with the recommended default parameters. To ensure that the identified STRs have high quality and were covered multiple times during the sequencing process, we filtered the STR loci using the lobSTR *filter vcf*. *py* script with the following parameters recommended in (Gymrek et al., 2012): loc-log-score = 0.8 (the minimum quality score for a locus to be included), loc-max-ref-length = 80 (the maximum reference allele needs to be at most 80 bps long), call-dist-end = 20 (the absolute value of the DISTENDS score should be at most 20), loc-call-rate = 0.8 (minimum call rate for a locus to be included is 80%), call-log-score = 0.8 (the minimum quality score for an STR call to be included). Additionally, we use the following stringent filters: loc-cov = 20 (each locus needs to be covered at least 20 times) and call-cov = 20 (each STR call needs to be covered at least 20 times). This way, we ensure that our catalog of wild orangutan STR variation comprises as few false positive STRs as possible.

### Absolute Short Tandem Repeat Dosage

In order to estimate the variation at a certain STR locus, a measure called STR dosage has been proposed in (Willems et al., 2014). The STR dosage is computed as follows: for hemizygous loci it is the number of base pairs by which the variant is different from the reference allele, whereas for heterozygous loci it is defined as the halved sum of the number of base pairs after subtracting the reference allele from each of the variants, hence the average difference. The STR dosage was shown to be correlated with the gene expression levels (Gymrek et al., 2015). Since we are rather interested in the extent of variation at a certain locus, more precisely in the contribution of each allele to the variability observed at a certain locus, as opposed to the directionality of variation (insertions or deletions with respect to the STR reference allele), we bring the following modification to the computation of the STR dosage described above. For both hemizygous and heterozygous loci, we took the absolute of dosage value. For instance, if we have the genotype 18 bps/22 bps and the reference STR is 20 bps long, the absolute STR dosage is (|18–20| + |22–20|)/2 = 2. If the genotype is 18 bps/18 bps and the reference STR is 24 bps long, the absolute dosage is |18–24| = 6.

### Expected Heterozygosity

We computed the expected heterozygosity (genetic diversity) of all autosomal STR loci which had at least two alleles different from the reference STR in each population (*Pongo abelii*, *Pongo pygmaeus*, *Pongo pygmaeus morio* and *Pongo pygmaeus*) using Nei’s unbiased estimate of heterozygosity per locus when the sample size is small (Nei, 1978):

$$H_{l}\;=\frac{2n}{2n-1}\left(1-\overset{a}{\underset{i=1}{\sum }}f_{i}^{2}\right)$$

where n is the number of individuals for whom the genotypes were available at that particular locus, a is the number of unique alleles at that locus, and fi is the frequency of the *i*th allele at that locus. For each population, we compute the unbiased mean heterozygosity per locus using Nei’s formula:

$$H=\sum_{l=1}^{N}H_{l}$$

where *N* is the number of loci considered.

### Genetic Differentiation

We computed the Rst as a measure of genetic differentiation of all autosomal STR loci which had at least two alleles different from the reference STR in each population (*Pongo abelii*, *Pongo pygmaeus*, *Pongo pygmaeus morio* and *Pongo pygmaeus*) using Slatkin’s estimate of popluation subdivision (Slatkin, 1995), analogous to Wright’s Fst (Wright, 1965) per locus when the sample size is small:

$$R_{\mathrm{ST}}\mathrm{= (S-}S_{W}\mathrm{)/S}$$

where S is the average squared difference in allele size between all pairs of alleles, and *S*_*W*_ is the average of the squares of differences in allele size within each subpopulation.

### STRUCTURE Analysis

We carried out a population structure analysis using the STRUCTURE software version 2.3.4, (Pritchard et al., 2000). We used all data from all 21 individuals on 116 loci, which were most heterozygous across the samples. We have used the following parameters: MAXPOPS = 3, BURNIN = 500000, NUMREPS = 1,000,000, with no prior population information, unphased genotypes, the admixture model and no linkage disequilibrium. These parameters have been shown to work best for human populations using STR data (Willems et al., 2014).

### Gene Ontology Enrichment Analysis

For each STR variant in our catalog, we identified its closest gene, i.e., the one for which the absolute distance from the start coordinate of the STR variant to the transcription start site (TSS) is the smallest. The coordinates of the transcription start sites of the *Pongo abelii* genes were retrieved from the Ensembl Genes section version 84 of the Biomart interface (Kinsella et al., 2011).

We performed a gene ontology study using the set of genes which have an STR variant situated at most 10 kb away from their transcription start site, where a large number of regulatory interactions occur (Neph et al., 2012). To follow a conservative approach, we restricted our analysis to the set of STR variants occurring in at least two individuals from one population.

We tested for gene ontology term overrepresentation within the identified human orthologs against the set of all orangutan genes which have gene ontology annotations, retrieved from the Biomart tool (Kinsella et al., 2011). The gene ontology enrichment tests were performed using the DAVID Bioinformatics Resources (Huang et al., 2009).

## Results

### Short Tandem Repeat Variation Patterns in the Wild Orangutan Populations

We genotyped STRs in whole-genome sequences of 21 orangutans from the Southeast Asian islands of Sumatra and Borneo. After imposing highly conservative filtering criteria on the set of STRs genotyped for each individual, our catalog of wild orangutan STR variation encompasses 70,594 STR variants and 415789 STR invariants genotyped across 137225 STR loci. Out of the total number of STR loci, a subset of 33,525 loci had length alteration in at least one individual with respect to the reference allele. This set of variant STR loci represents 24.4% of the total STR set. We found that both variant and invariant STR loci were enriched in dimers, more than 75% of both categories had a motif length of 2, in line with findings from human STRs (Willems et al., 2014), (Supplementary Figure S2).

First, we asked how many short tandem repeat variants an individual orangutan has on average. We discovered that an individual from the Sumatran population has on average 1954 *±* 1312 STR variants, whereas an individual from the Bornean population has on average 2,946 *±* 1846 STR variants. Since a Sumatran *Pongo abelii* genome serves as our reference, one can expect to observe fewer short tandem repeat gains and losses in a Sumatran individual compared to a Bornean one. Yet, we noticed another factor impacting our ability to genotype variants, which is the depth of the genome sequences. Indeed, we found that a genome’s sequencing depth is a highly strong predictor of the number of variant short tandem repeats we genotype, with a Spearman correlation coefficient of 0.90 (*p*-value = 5 *×* 10^*–*9^), see Supplementary Figure S1 for exonic and upstream STRs.

Searching for a variation measure that is not impacted by the genome coverage, we drew our attention to the extent of variation, instead of its absence/presence at a certain locus. To this end, we computed the absolute dosage (number of copies that are added or deleted) for each STR variant and then computed the mean number for each individual. We did not detect any correlation between the mean absolute dosage across an individual’s set of STR loci and how well-covered its genome was during the sequencing process (correlation coefficient = *−*0.079, *p*-value = 0.687).

**FIGURE 1:**
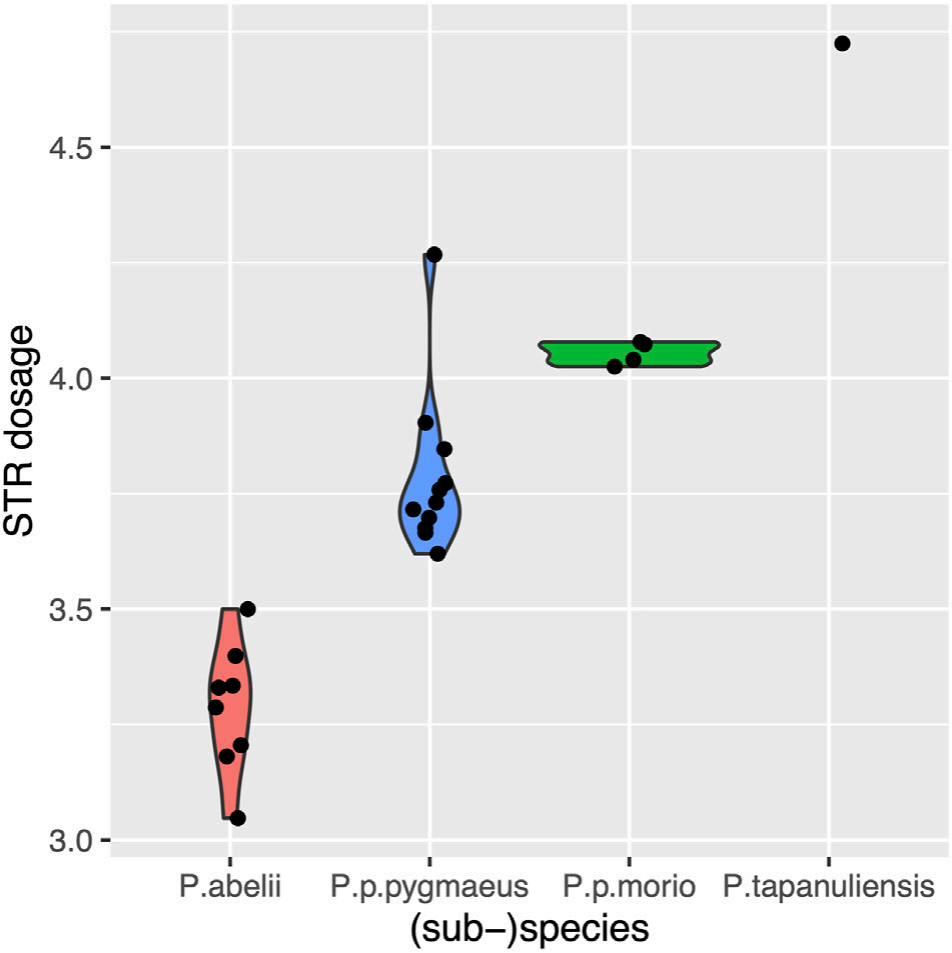
Violin plot for the mean absolute dosage of different (sub-)species. The mean values are 3.29 *±* 0.11, 3.79 *±* 0.12, 4.05 *±* 0.04, 4.72 for *P. abelii, P.p. pygmaeus, P. p.morio, P. tapanuliensis* (sub-)species respectively.

Once we found an approach to measure STR variation independent of genome quality, we got interested in species-specific variation we can detect with STRs. As Figure 1 depicts (sub-)species are clearly distinguished by their STR dosages. *P. abelii* from Sumatra, the species whose genome is the reference genome of the *Pongo* genus has the lowest dosage, with 3.29 *±* 0.11 nucleotides added or deleted. This is followed by *P. p.pygmaeus* and *P. p.morio*, two Bornean subspecies, which exhibit greater dosage 3.79 *±* 0.12*,* and 4.05 *±* 0.04 respectively. Finally, *P. tapanuliensis,* a geographically isolated species from Sumatra exhibits the greatest STR dosage with 4.72 nucleotides on average.

### Short Tandem Repeat Genetic Diversity and Differentiation Patterns

**FIGURE 2:**
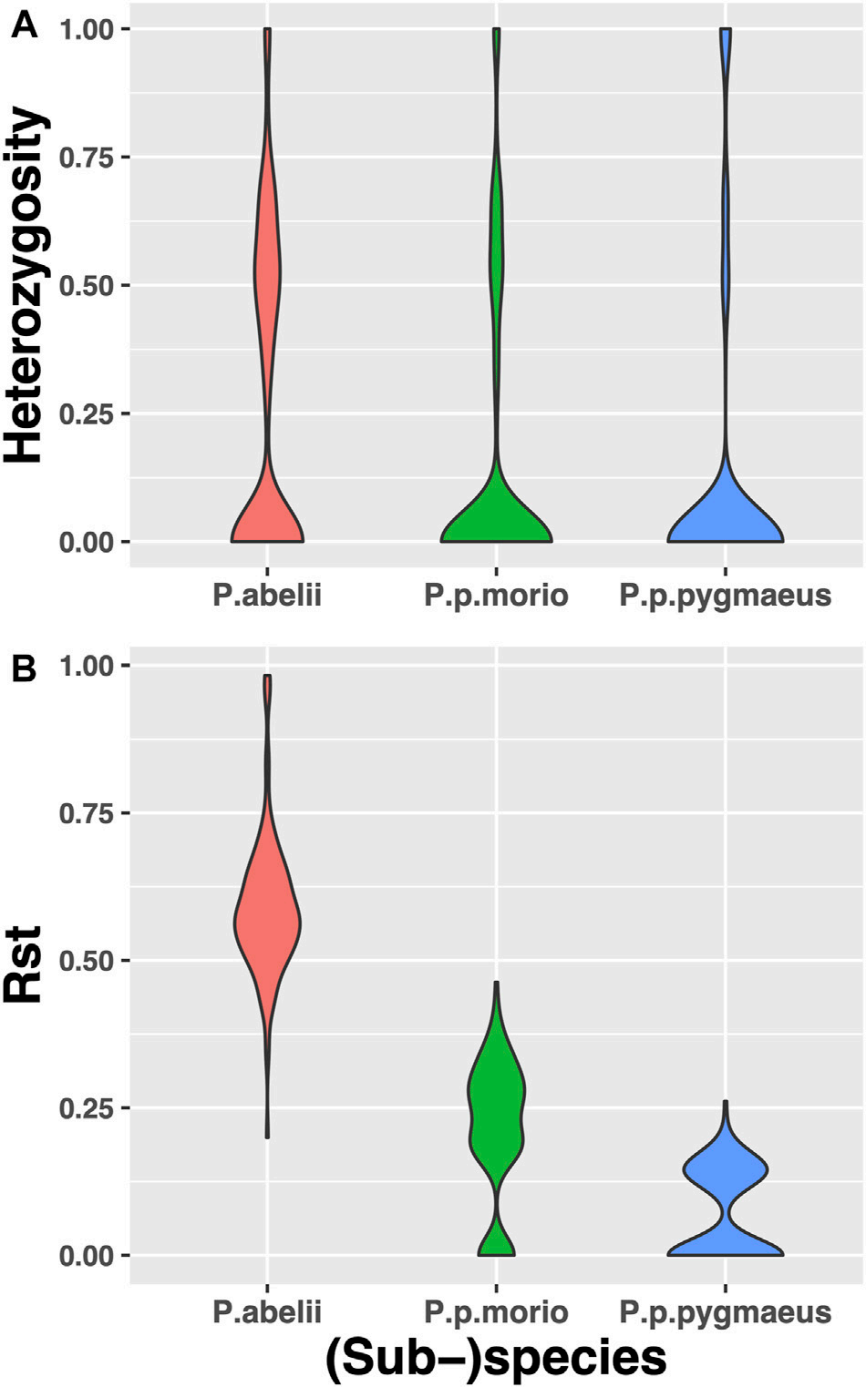
Expected Heterozygosity and Rst Violinplot Distributions based on STRs genotyped across 3 (sub-)species of *Pongo abelii*, *Pongo pygmaeus morio and Pongo pygmaeus*. *Pongo abelii* has the significantly highest expected heterozygosity and Rst and *Pongo pygmaeus* has the lowest.

We further explored the catalog of wild orangutan short tandem repeat variation by asking which population is more heterozygous. To this end, we identified 1287 STR loci which displayed variant alleles in the Sumatran *Pongo abelii* species, as well as in the two Bornean subspecies. We found that the mean heterozygosity for *Pongo abelii* is 0.258 ± 0.02, for *Pongo pygmaeus* is 0.121 ± 0.02, and for *Pongo pygmaeus morio* it is 0.144 ± 0.02. Pairwise Mann-Whitney U tests revealed that *Pongo abelii* has significantly higher gene diversity levels than *Pongo pygmaeus* (*p*-value *<* 2.2 *×* 10^*–*16^), and *Pongo pygmaeus morio* (*p*-value *<* 2.2 *×* 10^*–*16^) and *Pongo pygmaeus* has significantly lower gene diversity levels than *Pongo pygmaeus morio* (*p*-value = 2.199 *×* 10^*–*5^), see Figure 2A.

We then asked, whether we can correctly estimate the population differentiation based on our STR set. To this end, we calculated Rst values for each STR locus, which are analogous to Wright’s Fst, but assume a stepwise mutation model more in line with STR copy number evolution. We found that *P. abelii* has the greatest mean value of Rst (0.587), followed by *P. p.morio* (0.21) and *P. p.pygmaeus* (0.069), all of which are significantly different from each other based on Mann-Whitney U tests we performed with *p*-values smaller than 10^−16^ for each pair, see Figure 2B.

### Population Structure Based on Short Tandem Repeats

**FIGURE 3:**
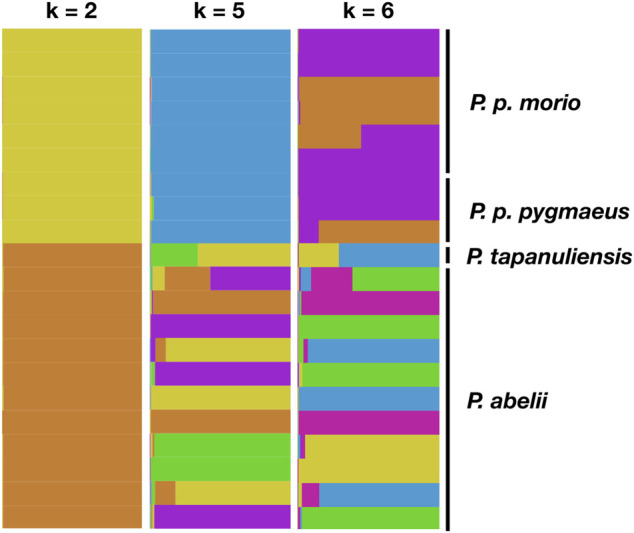
Bayesian clustering analysis of population structure of the genus *Pongo*. Each vertical line depicts an individual, with colors representing the inferred ancestry proportions with different assumed number of most distinctive genetic clusters of *k* = 2, 5, and 6 horizontally.

Next, we asked whether we can confirm the known population structure of the wild orangutan species using STRs. To this end, we used the software STRUCTURE, which performs clustering analysis using a Bayesian approach. We used a set of 116 loci, which were most heterozygous across the samples. Figure 3 presents the STRUCTURE maps for different assumed number of genetic clusters for *k* = 2, *k* = 5, and *k* = 6. We see an increase of different ancestry proportions in Sumatran samples, whereas Bornean samples present a rather unified ancestry in line with known population history of the genus based on SNP data (Locke et al., 2011; Prado-Martinez et al., 2013; Mattle-Greminger et al., 2018).

### Analysis of Short Tandem Repeat Variations Found in Promoter Regions

As repeats that are found in regulatory regions were shown to have an effect on phenotypic variation in great ape species (Bilgin Sonay et al., 2015; Sulovari et al., 2019), we decided to investigate the functions of the genes that are associated with STR mutations at possibly regulatory regions in wild orangutan genomes. To this end, we carried out a gene ontology enrichment study.

We identified a set of 2007 genes which have a variant short tandem repeat in their promoter region in at least two individuals in the Sumatran population. We discovered 2,358 such genes within the Bornean population.

Gene ontology enrichment for the Sumatran population unveiled three significantly overrepresented gene ontology terms belonging to the Biological Process category (see Table 1). These terms represent gene ontology terms related to the development of the nervous and skeletal system, as well as a term related to the renal system.

**TABLE 1:** List of GO terms in Sumatran and Bornean samples that are significantly enriched in genes close to variant STRs.

Origin	GO Term	# Of Genes	Enrichment	*p* Value (FDR)
**Sumatra**	Skeletal system development	11	3.4	1.4 × 10^−4^
**Sumatra**	Central Nervous System Development	10	3.6	2.0 × 10^−3^
**Sumatra**	Renal Water Homeostasis	5	6.7	6.3 × 10^−3^
**Borneo**	Positive regulation of ERK1 and ERK2 cascade	20	2.6	2.2 × 10^−4^
**Borneo**	Peptidyl-tyrosine phosphorylation	16	2.4	2.7 × 10^−3^
**Borneo**	Mammary gland alveolus gland	5	6.8	5.3 × 10^−3^
**Borneo**	Regulation of cell motility	6	4.9	6.5 × 10^−3^
**Borneo**	Phospholipase C- activating G-protein coupled receptor signaling pathway	9	3.1	7.5 × 10^−3^
**Borneo**	Protein Phosphorylation	13	2.4	8.6 × 10^−3^
**Borneo**	Cytokine Mediated Signaling Pathway	13	2.3	1.2 × 10^−2^
**Borneo**	Positive Regulation of Smooth Muscle Contraction	5	5.5	1.2 × 10^−2^

Gene ontology enrichment for the Bornean population uncovered eight significant terms belonging to the Biological Process category (see Table 1). Among the gene ontology terms enriched in the Bornean population, apart from the central signaling pathways, we can observe terms related to the reproductive system, such as cell motility and mammary gland development, as well as a term related to smooth muscle activity.

We carried out an additional Gene Ontology Enrichment Analysis using the human orthologs of the same set. This analysis unraveled many more GO terms uniquely enriched in Bornean and Sumatran genes that are close to a variant STR, which were clustered in the same functional categories. For Sumatran samples we found multiple terms involved in nervous system development and for Bornean samples, we listed terms related to cell motility and migration, as well as vasculature development. (see Supplementary Tables S2, S3 for details).

## Discussion

In this study, we explored the catalog of orangutan short tandem repeat variation to unveil patterns of variation, genetic diversity, as well as to assess the functional role of repeat variations within the wild orangutan population.

We first studied how the STR genotyping is impacted by a genome’s sequencing depth, for which we found a considerably high correlation of 0.90 (*p*-value = 5 *×* 10^*–*9^). Studies on human populations (Willems et al., 2014; Mousavi et al., 2019) showed that one can recover the true structure of human populations based on short tandem repeat loci genotyped genome-wide. Sufficient high-quality data combined with emerging PCR-free sequencing technologies make it possible to capture population demographic history based on genotyped STRs (Fungtammasan et al., 2015; Raz et al., 2019).

Because there are currently not as many high quality genomic data as there is for humans, instead of using the number of genotyped variants to study population variation, we use a measure called STR dosage proposed by (Willems et al., 2014). This measure quantifies the average allele change across an individual’s set of variant STR loci with respect to the reference set of short tandem repeats. We showed that the STR dosage is suitable for the assessment of the wild orangutan variation patterns, since it is independent of a genome’s sequencing depth.

Despite the low number of genomes that are have been sequenced from four (sub-)species (*P.abelii, P.p. pygmaeus, P.p.morio, P.tapanuliensis*), our STR-based population analyses accurately reflected their evolutionary distances to the reference species *P.abelii.* Consistent with its earliest split from the other Sumatran species approximately 3.4 million years ago (Nater et al., 2017), *P.tapanuliensis* exhibits the greatest STR dosage. Bornean species *P.p. pygmaeus, P. p.morio* follow that, in line with their migration to Borneo and split from orangutans in southern Sumatra migrated around 700 Kya (Mattle-Greminger et al., 2018). Remarkably, we were able to capture the more recent split around 10 Kya between two Bornean species, *P. p. pygmaeus* from mid-Borneo and *P. p.morio* from north Borneo. *P. p. pygmaeus* exhibits lower absolute dosage in line with its closer evolutionary distance to *P. abelii.*

Our genetic diversity analysis on the Sumatran and Bornean (sub-)species corroborated previous findings that the Sumatran populations are significantly more heterozygous than the Bornean populations (Prado-Martinez et al., 2013; Mattle-Greminger et al., 2018). Similarly, we observed in our STRUCTURE analysis a greater number of different ancestry populations in Sumatran samples compared to the Bornean ones. Such differences between the two islands have already been documented in the literature based predominantly on SNP markers (Locke et al., 2011; Prado-Martinez et al., 2013; Mattle-Greminger et al., 2018). Reduced genetic diversity levels are a consequence of several factors such as limited gene flow and high inbreeding that are prevalent for small-sized island populations (Furlan et al., 2012). Moreover, the Bornean populations may be taking longer to recover from their relatively recent bottleneck around 10 Kya (Mattle-Greminger et al., 2018). Also, Toba supereruption may have had an impact on the diversity levels within the wild orangutan populations since they may have triggered extinctions of the local species, which were followed by recolonizations of the affected areas (Nater et al., 2011; Ma et al., 2013). These factors likely influenced the Bornean population to a higher extent than the Sumatran one, especially the *P. pygmaeus* due to its smallest population size (Wich et al., 2008), thus rendering it less genetically diverse. Indeed, we found that the expected heterozygosity and the Rst estimates of *P. pygmaeus* are significantly lower than those of *P. p.morio*.

A recent study on human STRs found 28,000 STRs, for which repeat number is associated with expression of nearby genes (Fotsing et al., 2019). Indeed, one mechanism that STRs get involved in phenotypic divergence and adaptation is through gene regulation (Gemayel et al., 2010). In order to understand, if STR variations may be getting involved in local adaptations of wild orangutans, we assessed the potential impact of short tandem repeat variations on functional regions. Our gene ontology enrichment study revealed an enrichment of gene ontology terms related to nervous and skeletal system development as well as to kidney function in the Sumatran population. These terms and enriched terms obtained through our analysis on human orthologs of the orangutan genes are either the same or closely related terms as the enriched gene ontology terms for the Sumatran population reported in a SNP-based study (Mattle-Greminger et al., 2018). That study suggested a link between the observed enrichments terms related to neuronal development and the bigger brains and better cognitive skills Sumatran orangutans have compared to the Bornean ones (Taylor and van Schaik, 2007; Wich et al., 2009; Forss et al., 2016).

The gene ontology test for the Bornean population revealed enrichment for terms which relate to the cardiovascular system, such as vasculature development and smooth muscle activity. Enrichment of gene ontology terms related to heart activity has also been identified in a study investigating genes under selection in a genome-wide SNP survey, which suggested that the selection of such genes could be a response to the food shortage and temperature fluctuations experienced by the Bornean population during El Nino events (Mattle-Greminger et al., 2018).

To conclude, despite the low number of high-quality genomes that were available, using a highly polymorphic marker class of STRs, we were able to capture recent genetic diversity and adaptive history of a wild population at a great resolution (Nater et al., 2013; Mattle-Greminger et al., 2018). With greater number of better quality and PCR-free genomic sequences, we expect that further studies on wild species will reach the statistical power to detect many more novel variants linked to population structure and recent adaptive history. It is our belief that once repeat-compatible population and selection models become available, researchers will be able to infer STR evolution more accurately, resolving the issues that rise due to homoplasy (convergence of copy numbers due reverse mutations) and multi-allelic nature of STRs (Balloux and Lugon-Moulin, 2002). This way, we can hope to capture the demographic history of recently diverged populations at a much greater resolution, closing the gap caused by the missing heritability.

Furthermore, once ancient orangutan genome sequences are available, a time-series analysis of ancient and modern genomes may hopefully yield more insights in the *Pongo* evolutionary history, especially concerning the already extinct subspecies and their migration paths. Findings obtained by such population studies will be of special importance not only for orangutans but also for other critically endangered species which are under the threat of a possible sixth mass extinction due to abruptly changing environments (Ceballos et al., 2020).

## Data Availability

The datasets presented in this study can be found in online repositories. The names of the repository/repositories and accession number(s) can be found below: https://github.com/tbilgin/pongo_repeats.
