# Supplementary material for: Short Tandem Repeats as a High-Resolution Marker for Capturing Recent Orangutan Population Evolution
Source: Front Bioinform. 2021 Aug 16;1:695784. doi: 10.3389/fbinf.2021.695784 (PMC9581056; doi:10.3389/fbinf.2021.695784)
Supplement: Supplementary file 1 [file DataSheet1.pdf]

Supplementary Material

Species	Sampling Area	Individual ID	Depth	Source
P. abelii	Langkat	PA_A947	27.39	(Prado-Martinez et al., 2013)
P. abelii	Langkat	PA_A948	23.71	(Prado-Martinez et al., 2013)
P. abelii	Langkat	PA_A950	26.28	(Prado-Martinez et al., 2013)
P. abelii	Langkat	PA_A952	21.03	(Prado-Martinez et al., 2013)
P. abelii	North Aceh	PA_A949	27.39	(Prado-Martinez et al., 2013)
P. abelii	North Aceh	PA_B018	16.31	(Mattle-Greminger et al., 2018)
P. abelii	West Alas	PA_B017	17.78	(Mattle-Greminger et al., 2018)
P. abelii	West Alas	PA_A953	25.27	(Mattle-Greminger et al., 2018)
P. abelii	West Alas	PA_A955	11.06	(Mattle-Greminger et al., 2018)
P. abelii	West Alas	PA_A964	25.27	(Mattle-Greminger et al., 2018)
P. abelii	West Alas	PA_B020	16.30	(Mattle-Greminger et al., 2018)
P. tapanuliensis	Batang Toru	PA_B019	16.92	(Mattle-Greminger et al., 2018)
P. p. pygmaeus	Sarawak	PP_A939	20.48	(Prado-Martinez et al., 2013)
P. p. pygmaeus	Sarawak	PP_A942	23.12	(Mattle-Greminger et al., 2018)
P. p. pygmaeus	Sarawak	PP_A946	22.39	(Mattle-Greminger et al., 2018)
P. p. morio	East Kalimantan	PP_A984	29.89	(Mattle-Greminger et al., 2018)
P. p. morio	East Kalimantan	PP_A985	30.13	(Mattle-Greminger et al., 2018)
P. p. morio	South Kinabatangan	PP_5062	13.81	(Mattle-Greminger et al., 2018)
P. p. morio	South Kinabatangan	PP_A989	27.30	(Mattle-Greminger et al., 2018)
P. p. morio	North Kinabatangan	PP_A987	30.65	(Mattle-Greminger et al., 2018)
P. p. morio	North Kinabatangan	PP_A988	31.06	(Mattle-Greminger et al., 2018)

Table S1. Dataset of 21 whole genome sequences from four different (sub-)species of wild orangutans.

We computed the correlation between the number of genotyped variants and the genome's depth for STRs in promoter regions and in exonic regions as well, in order to see if the same correlation holds for those. We found those correlations to be significant, although lower (0.73, p-value = 1.6×10^{-4} and 0.51, p-value = 0.03 with FDR correction for STRs in exons and promoters respectively). Interestingly, *P.tapanuliensis,* a highly isolated species from Sumatra exhibits much more STR variants in its exons and promoters than other species. The much deeper split between the *P.tapanuliensis* and the others could be an explanation. Yet, because the difference only becomes visible in functionally relevant regions of the DNA, the selection may be acting on those mutated sites. With only one genome from this critically endangered species, we cannot come to a conclusion.

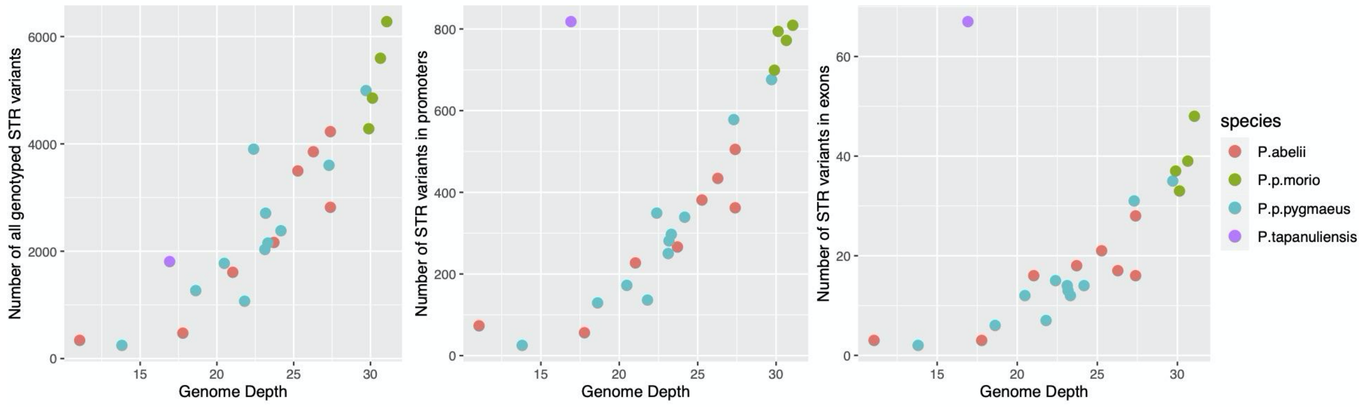

Figure S1: The number of STR variants per genome is strongly correlated with the genome's sequencing depth. The panels are for STRs genotypes throughout the genome, in promoters and in exons. The individuals are depicted according to their (sub-)species.

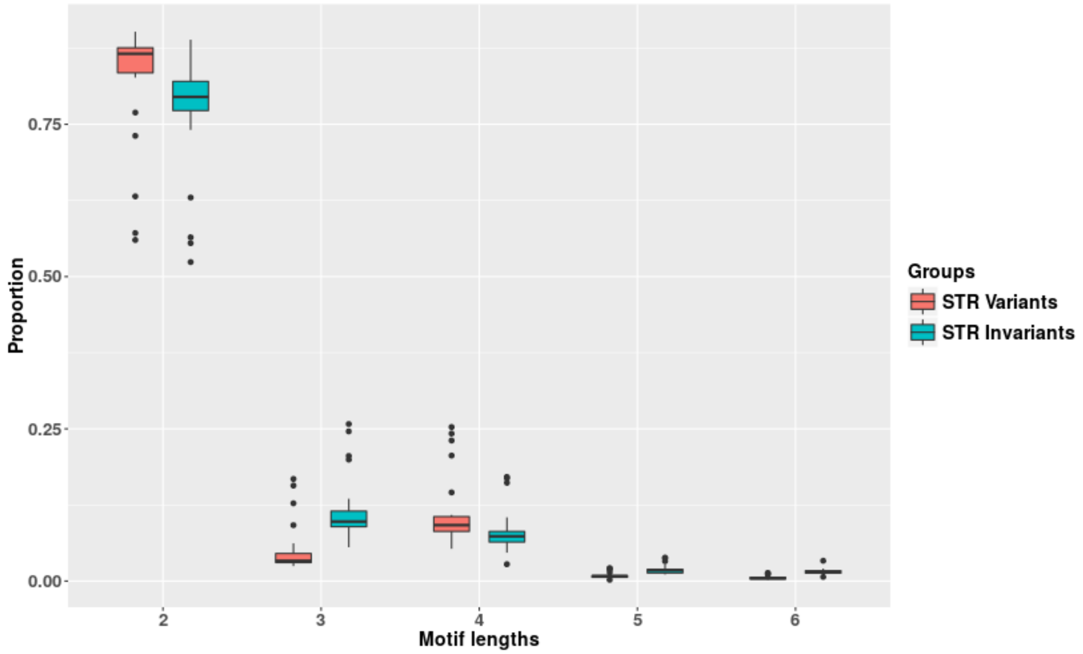

Figure S2. Boxplot distribution of STR motif lengths in the dataset. STRs that have length alteration in at least one individual are labelled as variants. The remaining ones are invariants.

Table S2. Gene Ontology Terms enriched uniquely in Sumatran populations for human orthologs.

GO.ID	Term	Annotated	Significant	Expected	classicFisher	fdrCorrection
GO:0009653	anatomical structure morphogenesis	2644	231	142.7	8.1E-15	1.176687E-10
GO:0048856	anatomical structure development	5216	388	281.52	3.2E-14	2.32432E-10
GO:0048731	system development	4363	335	235.48	9.6E-14	4.64864E-10
GO:0030030	cell projection organization	1253	130	67.63	2E-13	5.8108E-10
GO:0007399	nervous system development	2180	195	117.66	2.2E-13	5.8108E-10
GO:0030182	neuron differentiation	1229	128	66.33	2.4E-13	5.8108E-10
GO:0007275	multicellular organismal development	4964	366	267.92	1.2E-12	2.49034285714286E-09
GO:0048699	generation of neurons	1353	134	73.02	2.6E-12	4.721275E-09
GO:0022008	neurogenesis	1445	139	77.99	8E-12	1.29128888888889E-08
GO:0048666	neuron development	974	103	52.57	2.8E-11	4.06756E-08
GO:0031175	neuron projection development	823	91	44.42	4.2E-11	5.54667272727273E-08
GO:0048468	cell development	1983	173	107.03	5.6E-11	6.77926666666667E-08
GO:0032502	developmental process	5937	414	320.43	6.9E-11	7.71048461538462E-08
GO:0048858	cell projection morphogenesis	789	87	42.58	1.4E-10	1.35585333333333E-07
GO:0044767	single-organism developmental process	5842	407	315.31	1.4E-10	1.35585333333333E-07
GO:0032989	cellular component morphogenesis	1299	124	70.11	2.1E-10	1.90666875E-07
GO:0048646	anatomical structure formation involved ...	1186	115	64.01	4.1E-10	3.50357058823529E-07
GO:0032990	cell part morphogenesis	809	87	43.66	4.8E-10	3.87386666666667E-07
GO:0000902	cell morphogenesis	1215	116	65.58	8.6E-10	6.57537894736842E-07
GO:0048869	cellular developmental process	3974	291	214.49	2.4E-09	1.74324E-06
GO:0044707	single-multicellular organism process	7016	459	378.67	4.1E-08	2.83622380952381E-05
GO:0030154	cell differentiation	3688	267	199.05	4.8E-08	3.16952727272727E-05
GO:0048812	neuron projection morphogenesis	548	61	29.58	6.2E-08	3.91597391304348E-05
GO:0051960	regulation of nervous system development	755	76	40.75	1.1E-07	6.65820833333333E-05
GO:0048513	organ development	3194	235	172.39	1.2E-07	6.70476923076923E-05
GO:0032501	multicellular organismal process	7269	470	392.33	1.2E-07	6.70476923076923E-05
GO:2000026	regulation of multicellular organismal d...	1736	143	93.7	1.4E-07	7.53251851851852E-05
GO:0061564	axon development	447	52	24.13	1.5E-07	7.78232142857143E-05
GO:0021953	central nervous system neuron differenti...	164	27	8.85	2E-07	0.000100186206896552
GO:0000904	cell morphogenesis involved in different...	743	74	40.1	2.4E-07	0.000116216
GO:0048667	cell morphogenesis involved in neuron di...	506	56	27.31	2.6E-07	0.00012183935483871
GO:0060322	head development	720	72	38.86	3E-07	0.000136190625
GO:0051239	regulation of multicellular organismal p...	2745	205	148.15	3.4E-07	0.000149672121212121
GO:0072073	kidney epithelium development	140	24	7.56	4.5E-07	0.000192269117647059
GO:0007417	central nervous system development	910	85	49.11	4.7E-07	0.000195076857142857

**GO:0009888**	tissue development	1918	152	103.52	5.7E-07	0.000230010833333333
**GO:0007420**	brain development	683	68	36.86	7.5E-07	0.000294466216216216
**GO:0050793**	regulation of developmental process	2326	177	125.54	8.3E-07	0.000312889230769231
**GO:0001935**	endothelial cell proliferation	106	20	5.72	8.4E-07	0.000312889230769231
**GO:0072006**	nephron development	135	23	7.29	8.7E-07	0.00031596225
**GO:0045664**	regulation of neuron differentiation	548	57	29.58	1.5E-06	0.000531475609756098
**GO:0009790**	embryo development	985	88	53.16	1.9E-06	0.00065717380952381
**GO:0072080**	nephron tubule development	93	18	5.02	2E-06	0.000675674418604651
**GO:0060993**	kidney morphogenesis	94	18	5.07	2.4E-06	0.000789510869565217
**GO:0048589**	developmental growth	570	58	30.76	2.5E-06	0.000789510869565217
**GO:0048522**	positive regulation of cellular process	4958	331	267.6	2.5E-06	0.000789510869565217
**GO:0030902**	hindbrain development	144	23	7.77	2.7E-06	0.000834529787234043
**GO:0050767**	regulation of neurogenesis	668	65	36.05	2.9E-06	0.000877672916666667
**GO:0061326**	renal tubule development	96	18	5.18	3.2E-06	0.000939982352941176
**GO:0030900**	forebrain development	366	42	19.75	3.3E-06	0.000939982352941176
**GO:0072028**	nephron morphogenesis	78	16	4.21	3.3E-06	0.000939982352941176

Table S3. Gene Ontology Terms enriched uniquely in Bornean populations for human orthologs.

GO.ID	Term	Annotated	Significant	Expected	classicFisher	fdrCorrection
**GO:0009653**	anatomical structure morphogenesis	2644	219	143	1.5E-11	2.17905E-07
**GO:0048856**	anatomical structure development	5216	371	282.1	1.7E-10	1.11373666666667E-06
**GO:0048731**	system development	4363	320	235.97	2.3E-10	1.11373666666667E-06
**GO:0007275**	multicellular organismal development	4964	353	268.47	7.3E-10	2.6511775E-06
**GO:0044767**	single-organism developmental process	5842	401	315.96	2.4E-09	6.97296E-06
**GO:0032502**	developmental process	5937	406	321.1	2.9E-09	7.02138333333333E-06
**GO:0007399**	nervous system development	2180	177	117.9	1.1E-08	2.28281428571429E-05
**GO:0048646**	anatomical structure formation involved ...	1186	110	64.14	1.4E-08	2.542225E-05
**GO:2000026**	regulation of multicellular organismal d...	1736	147	93.89	1.8E-08	2.9054E-05
**GO:0009887**	organ morphogenesis	1019	97	55.11	3E-08	4.3581E-05
**GO:0048699**	generation of neurons	1353	119	73.18	6.6E-08	8.7162E-05
**GO:0048513**	organ development	3194	236	172.75	9.1E-08	9.65007857142857E-05
**GO:0048869**	cellular developmental process	3974	283	214.93	9.2E-08	9.65007857142857E-05
**GO:0050793**	regulation of developmental process	2326	182	125.8	9.3E-08	9.65007857142857E-05
**GO:0044707**	single-multicellular organism process	7016	457	379.46	1.2E-07	0.0001089525
**GO:0032501**	multicellular organismal process	7269	471	393.14	1.2E-07	0.0001089525
**GO:0022008**	neurogenesis	1445	124	78.15	1.3E-07	0.000111088823529412
**GO:0051239**	regulation of multicellular organismal p...	2745	207	148.46	1.7E-07	0.000137199444444444
**GO:0030154**	cell differentiation	3688	264	199.46	2E-07	0.000152915789473684
**GO:0010927**	cellular component assembly involved in ...	287	38	15.52	3.1E-07	0.0002251685

**GO:0048468**	cell development	1983	157	107.25	4.3E-07	0.000297457619047619
**GO:0030182**	neuron differentiation	1229	106	66.47	9.5E-07	0.000627302272727273
**GO:0009888**	tissue development	1918	151	103.73	1E-06	0.000631608695652174
**GO:0060322**	head development	720	70	38.94	1.3E-06	0.000786879166666667
**GO:0030900**	forebrain development	366	43	19.79	1.4E-06	0.000813512
